# Serine racemase expression profile in the prefrontal cortex and hippocampal subregions during aging in male and female rats

**DOI:** 10.18632/aging.205841

**Published:** 2024-05-17

**Authors:** Linda Bean, Prodip K. Bose, Asha Rani, Ashok Kumar

**Affiliations:** 1 Department of Anatomy, Cell Biology, and Physiology, IU School of Medicine, Indianapolis, IN 46201, USA; 2 Brain Rehabilitation Research Center, Malcom Randall Department of Veterans Affairs Medical Center, North Florida/South Georgia Veterans Health System, Gainesville, FL 32607, USA; 3 Department of Anesthesiology and Department of Neurology at the College of Medicine, University of Florida, FL 32607, USA; 4 Department of Neuroscience, The McKnight Brain Institute, University of Florida, Gainesville, FL 32607, USA

**Keywords:** aging, serine racemase, hippocampus, medial prefrontal cortex (mPFC), NMDA receptor

## Abstract

Aging is associated with a decrease in N-methyl-D-aspartate (NMDA) receptor function, which is critical for maintaining synaptic plasticity, learning, and memory. Activation of the NMDA receptor requires binding of the neurotransmitter glutamate and also the presence of co-agonist D-serine at the glycine site. The enzymatic conversion of L-serine to D-serine is facilitated by the enzyme serine racemase (SR). Subsequently, SR plays a pivotal role in regulating NMDA receptor activity, thereby impacting synaptic plasticity and memory processes in the central nervous system. As such, age-related changes in the expression of SR could contribute to decreased NMDA receptor function. However, age-associated changes in SR expression levels in the medial and lateral prefrontal cortex (mPFC, lPFC), and in the dorsal hippocampal subfields, CA1, CA3, and dentate gyrus (DG), have not been thoroughly elucidated. Therefore, the current studies were designed to determine the SR expression profile, including protein levels and mRNA, for these regions in aged and young male and female Fischer-344 rats. Our results demonstrate a significant reduction in SR expression levels in the mPFC and all hippocampal subfields of aged rats compared to young rats. No sex differences were observed in the expression of SR. These findings suggest that the decrease in SR levels may play a role in the age-associated reduction of NMDA receptor function in brain regions crucial for cognitive function and synaptic plasticity.

## INTRODUCTION

N-methyl-D-aspartate (NMDA) receptors are integral components in diverse physiological processes, exerting a crucial influence on synaptic plasticity, cognitive functions, psychiatric conditions, and the intricate connectivity of neural networks [1–6]. These receptors play a significant role in regulating signal transmission and information processing in the brain. Their involvement in synaptic plasticity underscores their importance in shaping the strength and efficiency of neuronal connections, which is pivotal for learning and memory. The age-associated decrease in NMDA receptor function profoundly influences both synaptic and cognitive function. [7–21]. The NMDA receptor requires the binding of glutamate and the co-agonist D-serine for activation [22–24]. Results from various investigations suggest that D-serine serves as the principal endogenous co-agonist for the activation of NMDA receptors within brain regions that are associated with cognition [25–27].

D-serine levels depend on serine racemase (SR), the enzyme that converts L-serine to D-serine [28–30]. Under physiological conditions, D-serine is produced by neuronal SR and is released postsynaptically [27, 31]. However, D-serine is also produced by reactive glia under inflammatory conditions such as injury and neurodegenerative disease [32–35]. D-serine availability modulates the maturation of neuronal circuitry and is involved in influencing various behavior measures [36, 37]. Previous studies have indicated a decrease in D-serine levels with aging, which was associated with alterations in glutamatergic synaptic transmission [26, 38–43]. Results demonstrate that an age-associated decrease of D-serine and its enzyme, SR, in the hippocampus [40, 41, 44, 45] and that NMDA receptor hypofunction in the hippocampus can be rescued by exogenous D-serine [40, 41, 46]. A recent study found that secreted amyloid protein precursor-α (sAPPα), derived from the cleavage of amyloid protein precursor, notably enhances NMDA receptor function exclusively in aged animals, restoring impaired long-term potentiation (LTP) associated with aging. Yet, this effect is significantly reduced in SR knockout mice [47]. Overall, the results suggest that D-serine is crucial for the activation of NMDA receptors and NMDA receptor-mediated synaptic plasticity. Therefore, it is conceivable that age-related alterations in the expression of SR may contribute to a reduction in NMDA receptor function.

Various brain regions exhibited similar age-related transcriptional changes. However, region-specific transcriptions were associated with the performance of cognitive tasks that depended on the corresponding brain region [48–50]. In studies concentrating on the three main hippocampal subregions, impaired spatial memory is correlated with substantial transcriptional differences in CA1 and CA3 regions but only minimal differences in the dentate gyrus (DG) [50–52]. The rodent dorsal hippocampus, corresponding to the human posterior hippocampus, is involved in the cognitive process of learning and memory associated with navigation, exploration, and locomotion [53]. Different hippocampal subregions (CA1, CA3, DG) contribute uniquely to memory, specializing in various aspects of formation, storage, and retrieval. This functional diversity enables the hippocampus to support complex mechanisms involved in spatial, episodic, and declarative memory. The unique properties and connectivity of each subregion contribute to the intricate and dynamic nature of the overall memory system in the hippocampus. Each subregion of the hippocampus has a unique contribution to the processing of memory including pattern separation, and pattern completion [54, 55]. Therefore, we further subdivided the dorsal hippocampus into CA1, CA3, and DG, and assessed the protein expression and mRNA levels in these areas. We also determined expression of SR in two additional regions: the ventral hippocampus (VH), which is involved with emotional behavior [53, 56] and the hypothalamus, a major link between the nervous system and the endocrine system which is also responsible for maintaining homeostasis [57]. Synonymous with previous research, we confirmed reduced protein and mRNA expression of SR in the aged rat hippocampus, with the greatest reductions seen in the CA3 and CA1 subregions. Interestingly, reduced SR expression was found in additional areas of interest including VH and hypothalamus. Our results of decreased SR expression in the VH with aging align with prior findings indicating reduced SR activity in the neuropil of the radial layer of the CA1 field in aged rats exposed to stress [58, 59].

Previous work from our lab suggested that an age-related decrease in NMDA receptor function in the medial prefrontal cortex (mPFC) contributes to impaired executive function in rodents [15]. Additionally, our recent results demonstrate that the viral vector-mediated increase in SR expression within the mPFC of middle-aged rats led to effective contingency acquisition in visual discrimination tasks, likely attributable to improved attentional function. In addition, electrophysiological recordings revealed a substantial enhancement in NMDA receptor-mediated synaptic responses recorded from the mPFC following the upregulation of SR expression [60]. Therefore, we asked whether SR could be reduced in the prefrontal cortex of our rat model. Results from previous studies have shown that there is no loss of SR in the cerebral cortex [41]. However, results were not delineated to specific subregions. As such, we isolated the prefrontal cortex into medial and lateral PFC (mPFC, lPFC) areas and performed Western blotting and reverse transcription-polymerase chain reaction (RT-PCR) assays on these subregions to access the expression levels of protein and mRNA respectively. Interestingly, we found a significant reduction of SR expression levels in both regions. These results reveal a decline in SR expression levels in the mPFC, lPFC, and all hippocampal subfields during aging. This decline could contribute to a reduction in NMDA receptor-mediated synaptic transmission and impaired cognition.

## MATERIALS AND METHODS

### Subjects

Young (4-6 months) and aged (22-26 months) male and female Fisher 344 rats were sourced from the National Institute on Aging via the University of Florida Animal Care and Service facility. All rats were pair-housed and maintained on a 12:12 h light cycle with ad libitum access to food and water. Before handling, rats were habituated to the facilities for at least one week. All experiments were conducted following the guidelines described by the US Public Health Service Policy on Humane Care and Use of Laboratory Animals and were approved by the University of Florida Institutional Animal Care and Use Committee.

### Tissue collection

Rats were weighed and anesthetized with isoflurane before decapitation. Brains were removed and the hypothalamus, left and right PFC, and left and right hippocampus were rapidly dissected on an ice-cold dish. Each half of the PFC was subdivided into medial (mPFC) and lateral (lPFC). Each hippocampus was first divided into dorsal and ventral (VH) areas. The dorsal hippocampus was further subdivided into CA1, CA3, and DG. All samples were flash-frozen in liquid nitrogen and stored at -80° C until processing for Western blotting or reverse transcription-polymerase chain reaction.

### Western blotting

For Western blot analysis, samples were sonicated in radio-immunoprecipitation assay (RIPA) buffer (Thermo Fisher, Waltham, MA, USA) supplemented with phosphatase and protease inhibitors, and ethylenediaminetetraacetic acid (EDTA) (Thermo Scientific). Lysates were centrifuged at 20,000 xg for 10 min at 4° C. Protein concentration was measured using a Pierce bicinchoninic acid assay (BCA) protein assay (Thermo Scientific Cat# 23227). Sample lysates were denatured in Laemmli buffer (BioRad, Hercules, CA, USA) containing 2-mercaptoethanol and boiled for 5 minutes. All samples and controls (10 μg/well) plus a standard were loaded into a 4-15% TGX-stain-free gel (Bio-Rad Cat# 5678085). Technical replicates (duplicates) were randomly positioned on the same gel. Following electrophoresis, gels were UV-activated (Bio-Rad ChemiDoc) for 1 minute prior to transferring to LF-PVDF membranes using the Trans-Blot Turbo RTA transfer kit and Transfer System (Bio-Rad). Membranes were imaged for Total Protein (Bio-Rad ChemiDoc) prior to blocking with Intercept blocking buffer (LI-COR, Cat# 927-60001). Membranes were probed for SR antibody (Santa Cruz sc-365217, 1:1000) and β-actin (Abclonal AC026, 1:10,000) overnight at 4° C. Li-Cor near-infrared secondary antibodies (IRDye 800CW 1:20,000 and IRdye 680LT 1:10,000) were applied for 1 hour at room temperature. Membranes were washed with tris-buffered saline with tween (TBST) and tris-buffered saline (TBS) before scanning on the Odyssey CLx Infrared Imaging System (LI-COR Biosciences, Lincoln, NE, USA). Sample bands were quantified in Image Studio Lite Ver 5.2 (LI-COR Biosciences). Total Protein (range 30kDa-100kDA) was quantified using Image Lab Software Ver 6.1 (Bio-Rad Laboratories). Raw data was combined in Excel (Microsoft). Raw signals were first normalized to a Total Protein Lane Normalization Factor (LNF) and technical replicates were averaged for each animal. The means were then used to calculate the fold increase/decrease over young control per blot. Experiments were repeated at least twice or more times with sample position (lane) randomized between blots. To compare across blots, the control master mix, which contained equal concentrations of young protein lysate, was loaded onto every blot. Results from independent experiments were combined, and the mean, SD, and inter-assay %CVs were calculated for each animal. Values are reported as fold-change from the young control group (young=1.000). In addition, signals were also normalized to B-actin housekeeping protein (HKP) for comparison with values obtained by the Stain-Free Total Protein normalization method. Discrepancies between the data obtained by utilization of the two normalization methods are reported where they occur.

### Reverse transcription-polymerase chain reaction (RT-PCR)

RNA was isolated using the RNeasy Lipid Tissue Mini kit (Qiagen, Cat#74804), and DNase digestion was performed with the RNase-Free DNase set (Qiagen, Cat#79254). The concentration was measured with a NanoDrop 2000 spectrophotometer. For Reverse Transcription Quantitative Polymerase Chain Reaction (RT-qPCR), cDNA was prepared using the QuantiTect Reverse Transcription kit (Qiagen, Cat#205311) following the manufacturer’s protocol. Gene expression was quantified using TaqMan Gene Expression Assay for Serine racemase (SRR) (TaqMan Assay ID: Rn01648369_m1, Cat# 4331182, Applied Biosystems, Foster City, CA, USA) in a QuantStudio3 Applied Biosystems as per the manufacturer’s instructions. Samples were loaded in triplicate. The ΔΔCT method [61] was used to determine the relative change in gene expression levels. Values were normalized to beta (β)-actin (ACTB gene) and experiments were run in duplicate.

### Statistical analysis

For statistical analysis, Statview software was used to perform a one-way analysis of variance (ANOVA) to indicate significant differences in SR expression levels between young (YA) and aged (OA) rats. *Post hoc test* was used to uncover specific differences between group means when an analysis of variance test is significant. Data were interpreted as statistically significant if *p*≤0.05.

## RESULTS

### SR protein was decreased in the male rat brain.

**Figure 1:**
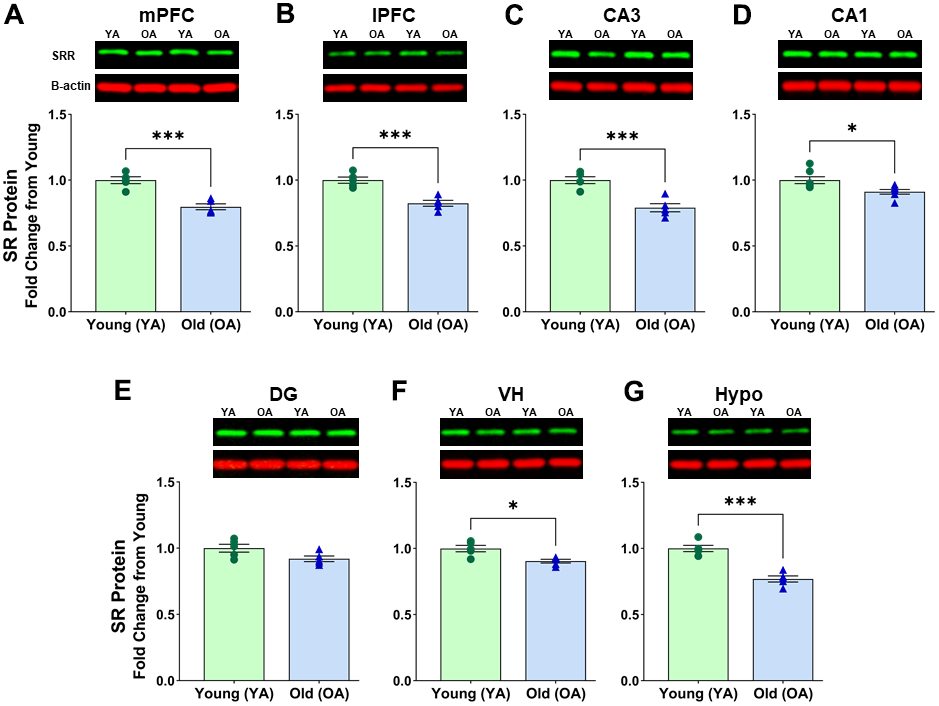
**Protein levels of serine racemase were decreased with age in the male F344 rat brain.** Western blots demonstrating expression of SR in (**A**) medial prefrontal cortex (mPFC), (**B**) lateral prefrontal cortex (lPFC), (**C**) CA3 subfield of the hippocampus, (**D**) CA1 subfield of the hippocampus, (**E**) Dentate gyrus (DG) subfield of the hippocampus, (**F**) ventral hippocampus (VH), (**G**) hypothalamus (Hypo). Bar graphs illustrate the quantitative analysis of immunoreactivity for SR when normalized to total protein. B-actin is shown for visual comparison only. Blots for total protein can be seen in Supplementary Figure 1. Asterisks for *p*-values indicate significance (*p<0.05, **p<0.005, ***p<0.0005).

First, we compared the protein expression of SR in subregions of the PFC between aged (OA, 26 mo) and young (YA, 5 mo) male Fischer 344 rats. There was a significant effect of age on SR protein levels in mPFC [F(1, 8) = 35.542, *p*=0.0004] (Figure 1A and Supplementary Figure 1A) and lPFC [F(1, 8) = 29.872; *p*=0.0006] (Figure 1B and Supplementary Figure 1B). *Post hoc* test indicated that the mean value of SR protein in the mPFC was significantly reduced (*p*< 0.0005, n = 5/age) in aged rats (M=0.798, SD=0.051, %CV=7.4) when compared to young rats (M=1.000, SD=0.059, %CV=3.4). The mean values of SR protein expression in the lPFC were reduced (*p* <0.001, n=5/age) in aged male rats (M=0.824, SD=0.049, %CV=6.9) compared to young male rats (M=1.000, SD=0.052, %CV=5.1). These results suggest a decline in SR protein levels with advanced age within the two subregions of the PFC in male rats.

Next, we compared the protein expression of SR in subregions of the hippocampus. ANOVAs across the hippocampal subfields CA3 [F(1, 8) = 26.554; *p* =0.0009] (Figure 1C and Supplementary Figure 1C) and CA1 [F(1, 12) = 8.108; *p* =0.0147] (Figure 1D and Supplementary Figure 1D) suggested a significant difference in SR protein levels in hippocampal subregions. The mean values of SR protein expression showed a significant decrease of SR protein levels in CA3 (*p* <0.001, n=5/age), and in CA1 (*p* <0.05, n=7/age), of aged male rats when compared to young male rats (CA3 aged: M=0.791, SD=0.069, %CV=6.5; CA3 young: M=1.000, SD=0.059, %CV=6.3; CA1 aged: M=0.912, SD=0.055, %CV=4.5; CA1 young: M=1.000, SD 0.069, %CV=4.2). Interestingly, there was only a trend for an effect of age on SR protein levels in DG [F(1, 8) = 4.338; *p* =0.0590], (DG aged: M=0.920, SD=0.047, %CV=7.0; DG young: M=1.000, SD=0.066, %CV=7.5) (Figure 1E and Supplementary Figure 1E). For all male rat subregions, similar Western Blot results were obtained when signals were normalized to β-actin, except for the DG. For the DG samples, further analysis showed that the housekeeping protein β-actin normalized to total protein was significantly different between aged and young [F(1, 8) = 5.930; *p* < 0.05] with 3 of the 5 aged animals contributing to this difference. Therefore, caution must be used when interpreting protein levels from the DG of young and old animals when protein is normalized to the B-actin housekeeping protein due to variability in older animals.

Additionally, we found a significant effect of age in the male VH [F(1, 8) = 13.799; *p* =0.0059] (Figure 1F and Supplementary Figure 1F) and the male hypothalamus [F(1, 8) = 40.607; *p* =0.0002] (Figure 1G and Supplemental Figure 1G). *Post hoc* tests indicated a decline of SR in the VH (*p* <0.01, n=5/age) of aged male rats (M=0.892, SD=0.036, %CV=4.3) when compared to young males (M=1.000, SD=0.054, %CV=5.6), and a decline of SR in the hypothalamus (*p* <0.0005, n=5/age) of aged male rats (M=0.784, SD=0.055, %CV=7.0) compared to the young males (M=1.000, SD=0.053, %CV=8.8).

### SR mRNA expression was reduced in the male rat brain

**Figure 2:**
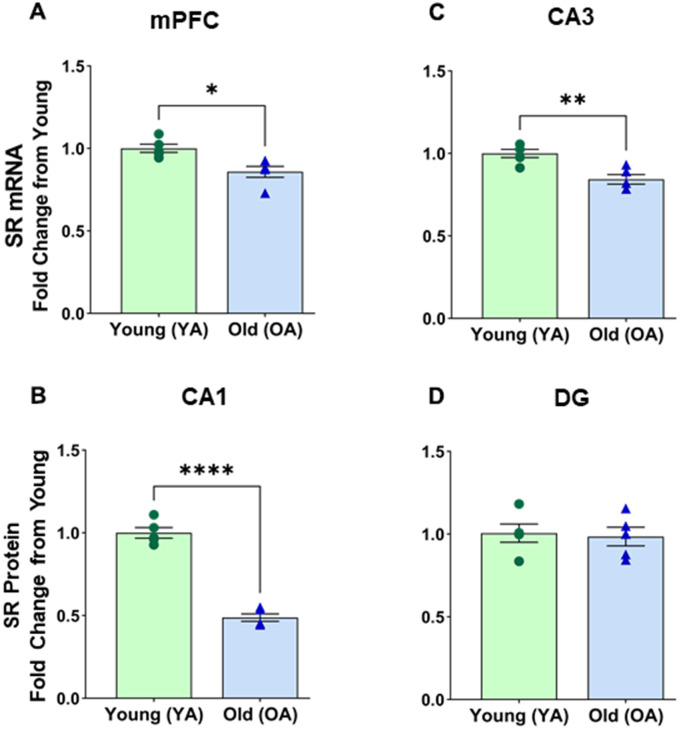
**mRNA levels of serine racemase, as determined by RT-PCR analysis, decreased with age in select subregions of the male Fisher 344 rat brain.** Bars demonstrating the quantitative fold change in SR mRNA in (**A**) medial prefrontal cortex (mPFC), (**B**) CA1 subfield of the hippocampus, (**C**) CA3 subfield of the hippocampus, (**D**) No changes in dentate (DG) subfield of the hippocampus were observed. Asterisks for *p*-values indicate significance (*p<0.05, **p<0.005, ***p<0.0001).

Given the age-related decrease in SR protein expression, we decided to examine mRNA alterations in a few specific regions, namely the medial prefrontal cortex (mPFC), CA1, CA3, and DG. A notable age-related effect was observed on SR mRNA expression in the mPFC [F(1, 8) = 11.177, p = 0.0102] (Figure 2A). *Post hoc* tests confirm a decline of SR mRNA in the mPFC (*p* <0.05, n=5/age) of aged male rats (M=0.858, SD=0.075) compared to the young males (M=1.000, SD=0.058). There was a significant effect of age on SR mRNA expression in CA1 [F(1, 8) = 170.345, *p* <0.0001] (Figure 2B). *Post hoc* tests confirm a decline of SR mRNA in the CA1 region (*p* <0.0001, n=5/age) of aged male rats (M=0.488, SD=0.051) compared to the young males (M=1.000, SD=0.071). There was a significant effect of age on SR mRNA expression in CA3 [F(1, 8) = 16.832, *p* =0.0034] (Figure 2C). *Post hoc* tests confirm a decline of SR mRNA in the CA3 region (*p* <0.005, n=5/age) of aged male rats (M=0.843, SD=0.065) compared to the young males (M=1.000, SD=0.056). No significant differences were found in SR mRNA expression between young and aged male DG [F(1, 8) = 0.068, p=0.8012] (Figure 2D).

### SR protein expression was reduced in the female rat brain

**Figure 3:**
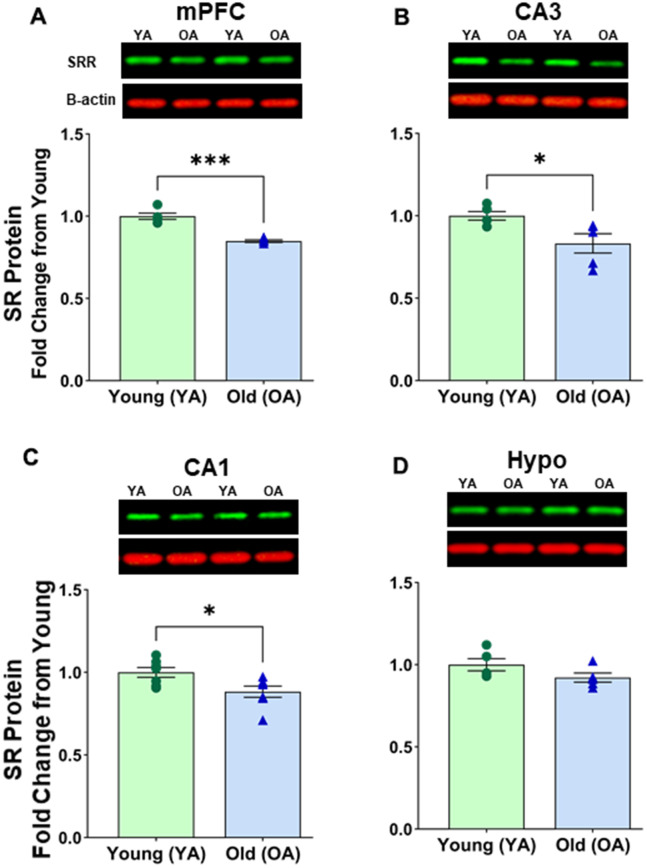
**Protein levels of serine racemase were reduced with age in select areas of the female Fisher 344 rat brain.** Western blots demonstrating expression of SR in (**A**) medial prefrontal cortex (mPFC) (**B**) CA3 subfield of the hippocampus, (**C**) CA1 subfield of the hippocampus, (**D**) hypothalamus (Hypo). Bar graphs illustrating the quantitative analysis of immunoreactivity for SR when normalized to total protein. B-actin is shown for visual comparison only. Blots for total protein can be seen in Supplementary Figure 2. Asterisks for *p*-values indicate significance (*p<0.05, ****p<0.0001).

We compared SR protein expression in specific brain regions of old female (22-26 months) and young female (~6 months) rats. In the female mPFC, a significant age-related effect was observed [F(1, 8) = 54.889, p < 0.0001] (Figure 3A and Supplementary Figure 2A). *Post hoc* test indicated that the mean value of SR expression in mPFC was significantly reduced (*p* < 0.0001, n = 5/age) in aged female rats (M=0.849, SD=0.017, %CV=7.8) when compared to young female rats (M=1.000, SD=0.042, %CV=6.2). In the hippocampus, there was also a significant effect of age on SR expression in the female rat CA3 [F(1, 8) = 6.865, p=0.0306] (Figure 3B and Supplementary Figure 2B) and the female CA1 [F(1, 12) = 6.763, p=0.0232] (Figure 3C and Supplementary Figure 2C). *Post hoc* test indicated that the mean value of SR expression was significantly reduced in the CA3 (*p* < 0.05, n = 5/age) in aged female rats (M=0.833 SD=0.131, %CV=8.4) when compared to young female rats (M=1.000, SD=0.057, %CV=5.9). In addition, there was a significant reduction in the CA1 (*p* < 0.05, n = 7/age) in aged female rats (M=0.884 SD=0.090, %CV=8.5) when compared to young female rats (M=1.000, SD=0.077, %CV=5.9). However, we did not see a significant change in the hypothalamus [F(1, 8) = 2.895, p=0.1273] (Figure 3D and Supplementary Figure 2D).

### SR protein expression in the mPFC during aging and sexual dimorphism

**Figure 4:**
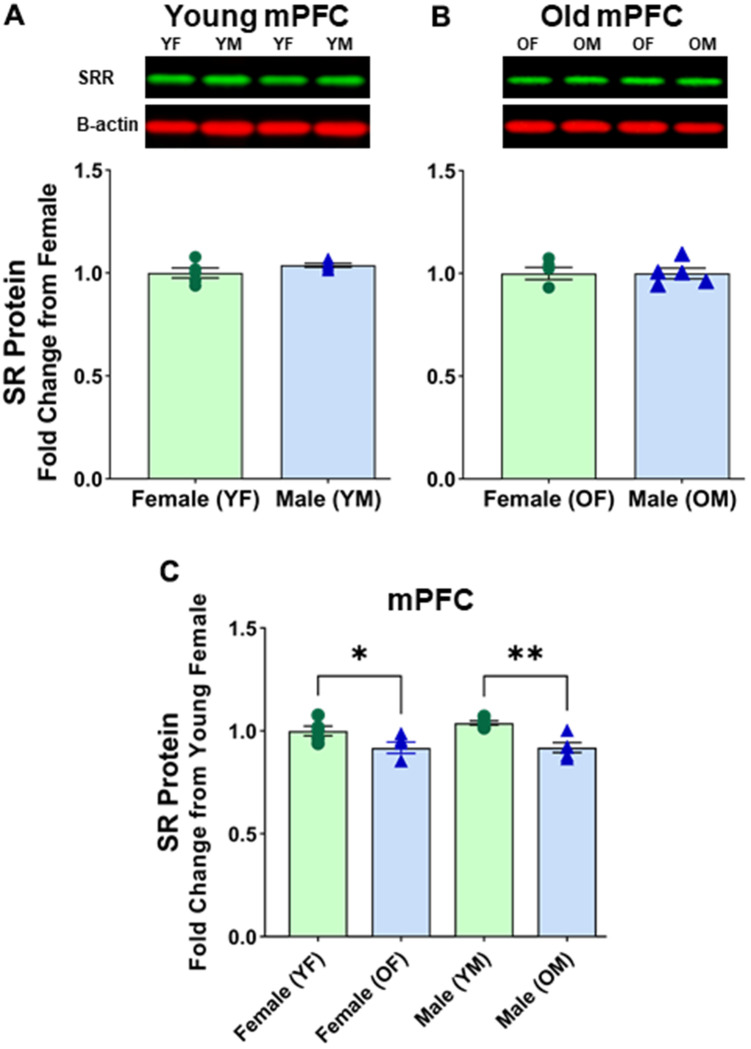
**No sex differences in protein levels of serine racemase in the mPFC were observed.** Western blots demonstrating expression of SR in (**A**) young female vs young male, and (**B**) old female vs old male. For A and B, fold changes were calculated from females for each age group. (**C**) depicts fold change of protein levels adjusted from young females. Bar graphs depict quantitative analysis of immunoreactivity for SR when normalized to total protein (see supplementary figure 3). The signal for B-actin is shown here for visual comparison only.

We wanted to see if there was a difference between males and females in the mPFC region, which is known for its susceptibility to NMDA receptor malfunctions during aging. As expected, no significant differences were found between young female and young male mPFC [F(1, 8) = 2.121, p=0.1834] (Figure 4A and Supplementary Figure 3A) or between aged female and aged male mPFC [F(1, 8) = 2.858E4, p=0.1834] (Figure 4B and Supplementary Figure 3B). Interestingly though, there was an indication of significance between aged female mPFC and aged male mPFC when normalizing to B-actin [F(1, 8) = 8.336, p=0.0203]. *Post hoc* test indicated that the mean value of SR expression was significantly less for aged males (M=0.808, SD=0.121, %CV=5.8) vs aged females (M=0.939, SD=0.121, %CV=8.2) (*p* < 0.05, n = 5/age). However, further analysis revealed that B-actin normalized to total protein was significantly different between aged female vs aged male mPFC [F(1, 8) = 5.961, p=0.0405] (Aged female M=0.892, SD=0.082; %CV=3.1; Aged male M=1.000, SD=0.024, %CV=15.9). The disparity between normalization methods suggests that using β-Actin as the housekeeping protein may not yield reliable results when comparing aged males and females.

## DISCUSSION

The findings of the present study reveal that aging is linked to a decline in SR protein levels across various brain regions, including the prefrontal cortex (both medial and lateral PFC), dorsal hippocampal subregions (CA3 and CA1), ventral hippocampus, and hypothalamus. Additionally, we observed a reduction in SR mRNA levels associated with advancing age in the mPFC, CA1, and CA3. Intriguingly, there was no decrease in SR mRNA in the DG, despite a discernible trend toward reduced SR protein levels in this subfield. Notably, we encountered variability in the expression of the B-actin housekeeping protein in the DG, prompting consideration for a reanalysis of mRNA expression using an alternative loading control. In parallel with the observations in male rats, our investigation of the aged female rat brain revealed a noteworthy decrease in SR protein levels within the mPFC and the CA3 and CA1 subfields of the hippocampus. Interestingly, in contrast to the male counterparts, we did not observe a significant reduction in SR protein levels within the hypothalamus of age and young females. It is worth noting that our study did not account for the time interval between the loss of ovarian function and the point of sacrifice. Consequently, it is conceivable that some of the aged female rats may exhibit lingering effects of estrogen, potentially manifesting as a form of neuroprotection within this specific brain region. This raises the possibility that the observed differences in SR protein levels in the aged female hypothalamus could be influenced by the temporal aspects of hormonal changes, warranting further investigation into the intricate interplay between estrogen fluctuations and SR expression in the aging female brain. When comparing the mPFC between young males and young females, as well as between aged males and aged females, we observed no significant differences.

Interestingly, our findings corroborate with previous studies that reported the loss of SR protein and mRNA in the hippocampus [40, 41], but diverge in revealing differences in both the medial and lateral PFC. In contrast to other studies that found no difference in the cerebral cortex of aged male Wistar rats [41], our focus on the PFC highlighted a decline in SR levels with aging in this region. This observation is crucial as the mPFC, analogous to the human dorsolateral PFC, governs executive functions including attention and cognitive flexibility [62–65]. NMDA receptor hypofunction in this region has been linked to reduced attention and learning in aged rats [15]. The decrease in SR protein, and consequently, the reduction in D-serine, may directly contribute to the loss of NMDA receptor function.

Additionally, these findings suggest the absence of sexual dimorphism in SR expression within this particular brain region. Additionally, our findings, revealing a decline in SR expression in the hippocampal subregions with aging, are consistent with previous research indicating a reduction in SR activity in the neuropil of the radial layer of the CA1 field in aged rats exposed to stress [58, 59]. This alignment suggests a potential connection between age-related changes in SR expression and stress-induced alterations in hippocampal regions. Moreover, our results extend this observation to encompass other hippocampal subregions, strengthening the link between decreased SR expression and age-related changes in hippocampal function, particularly in contexts involving stress exposure.

The findings from our recent study illustrate that the viral vector-mediated upregulation of SR expression in the mPFC of middle-aged rats led to effective contingency acquisition during visual discrimination, suggesting a potential improvement in attentional function. Moreover, electrophysiological recordings revealed a significant enhancement in NMDA receptor-mediated synaptic responses recorded from the mPFC as a result of the upregulation of SR expression. These observations suggest a link between the increased expression of SR, improved attentional function, and enhanced NMDA receptor-mediated synaptic responses, shedding light on the potential neurobiological mechanisms underlying the observed behavioral effects [60]. The present findings provide experimental support for the hypothesis positing that the reduction in SR expression may be a contributing factor to the decline in NMDA receptor function and potentially exert a negative influence on cognitive function.

The observed indirect correlation between decreased SR expression in mPFC and hippocampal subregions and NMDA receptor activity implies a potential role of SR in modulating cognitive processes. These results contribute to our understanding of the intricate relationship between SR expression and NMDA receptor function, shedding light on a potential mechanism underlying cognitive decline. Further investigations into the molecular and functional aspects of this association may yield valuable insights for developing targeted interventions to mitigate cognitive deficits associated with alterations in SR expression.

## Supplementary Material

Supplementary Figures
