# Supplementary material for: Serine racemase expression profile in the prefrontal cortex and hippocampal subregions during aging in male and female rats
Source: Aging (Albany NY). 2024 May 17;16(10):8402–16. doi: 10.18632/aging.205841 (PMC11164512; doi:10.18632/aging.205841)
Supplement: Supplementary Figures [file aging-16-205841-s001.pdf]

SUPPLEMENTARY FIGURES

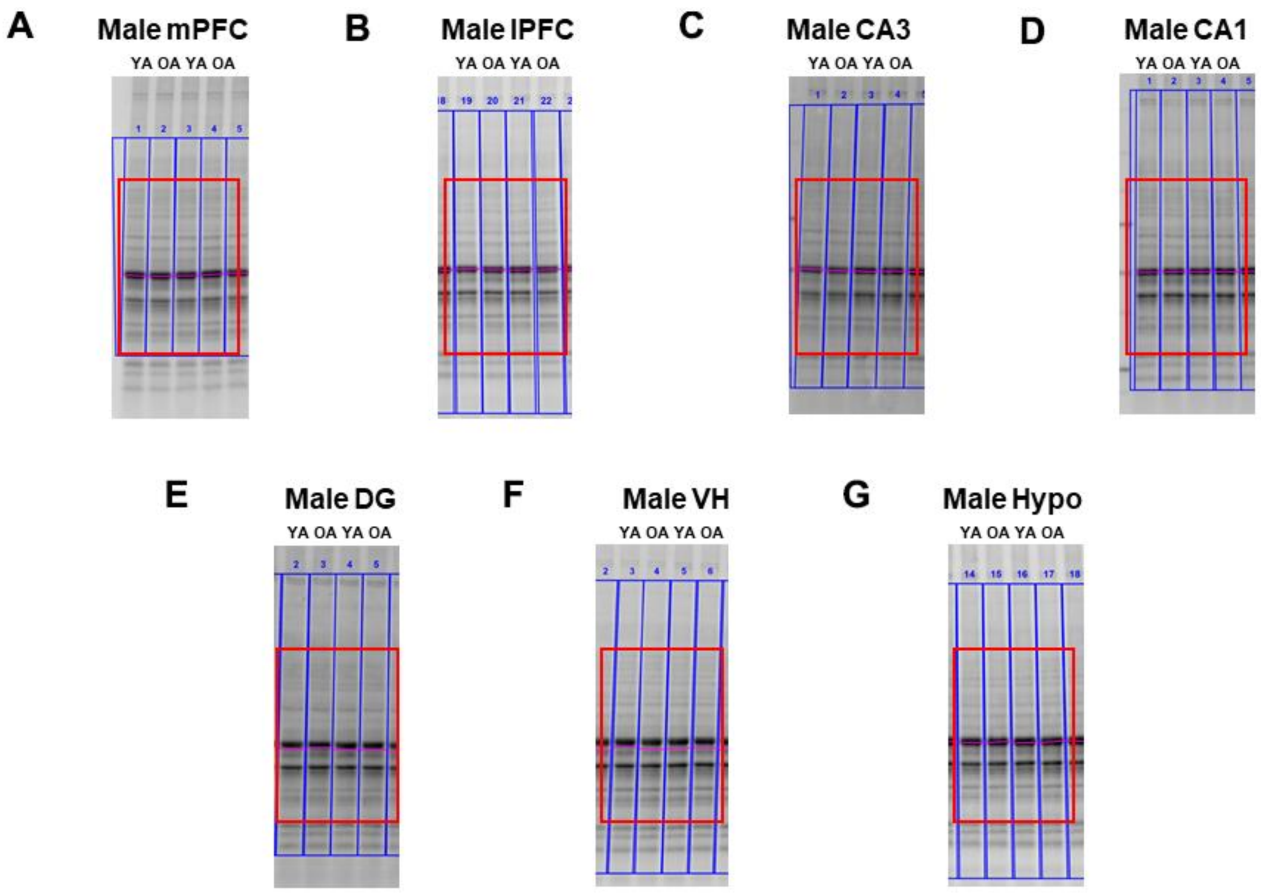

Supplemental Figure 1. Representative images for Total Protein correspond to the same bands/lanes seen in Figure 1 for young (YA) and old (OA) male rats. Western blots demonstrating expression of Total Protein in (**A**) medial prefrontal cortex (mPFC), (**B**) lateral prefrontal cortex (lPFC), (**C**) CA3 subfield of the hippocampus, (**D**) CA1 subfield of the hippocampus, (**E**) Dentate gyrus (DG) subfield of the hippocampus, (**F**) ventral hippocampus (VH), and (**G**) hypothalamus (Hypo). The blue lines represent the designated lanes, while the red boxes indicate the measured area (from low molecular weight to high molecular weight). These measurements are consistent with previous studies, where the authors measured total protein [1].

REFERENCES

1. Kirshner ZZ, Gibbs RB. Use of the REVERT® total protein stain as a loading control demonstrates significant benefits over the use of housekeeping proteins when analyzing brain homogenates by Western blot: An analysis of samples representing different gonadal hormone states. Mol Cell Endocrinol. 2018; 473:156–65. https://doi.org/10.1016/j.mce.2018.01.015 PMID:29396126

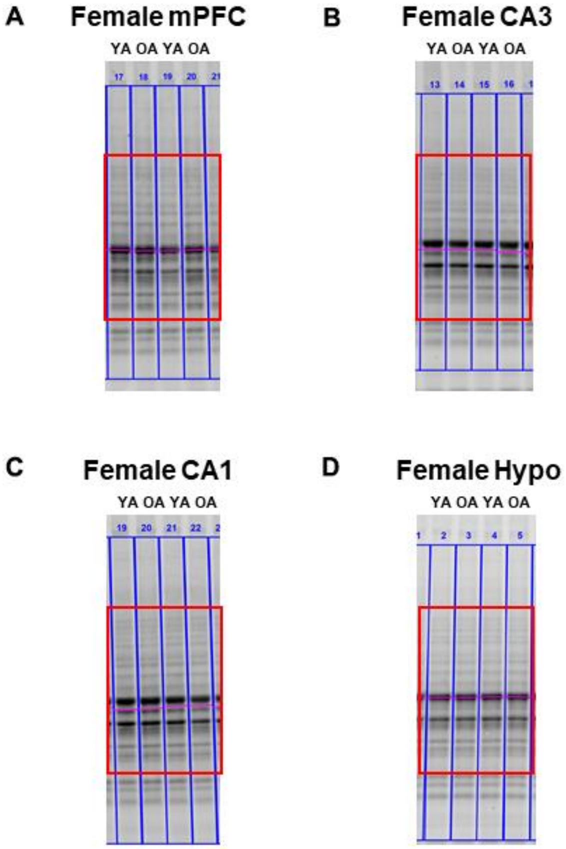

Supplemental Figure 2. Representative images for Total Protein correspond to the same bands/lanes seen in Figure 3 for young (YA) and old (OA) female rats. Western blots demonstrating expression of Total Protein in (**A**) medial prefrontal cortex (mPFC), (**B**) CA3 subfield of the hippocampus, (**C**) CA1 subfield of the hippocampus, and (**D**) hypothalamus (Hypo). The blue lines represent the designated lanes, while the red boxes indicate the measured area (from low molecular weight to high molecular weight).

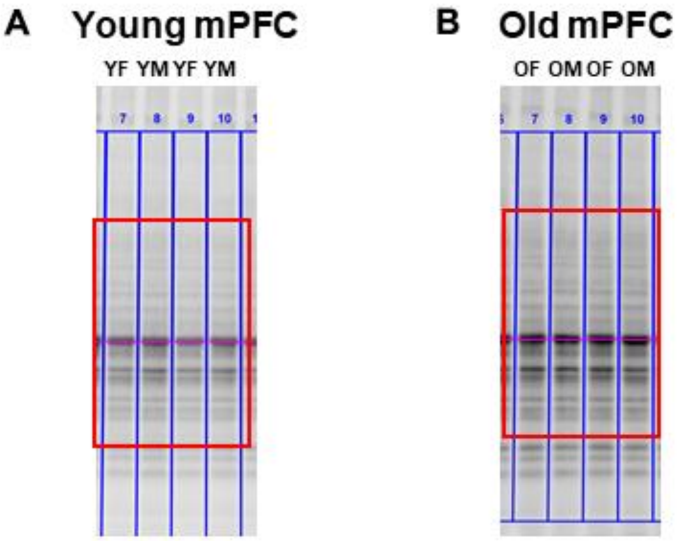

Supplemental Figure 3. Representative images for Total Protein correspond to the same bands/lanes seen in Figure 4 for young females (YF) and young males (YM), and old females (OF) and old male rats (OM). Western blots demonstrating expression of Total Protein in (**A**) young female vs young male, and (**B**) old female vs old male. The blue lines represent the designated lanes, while the red boxes indicate the measured area (from low molecular weight to high molecular weight).
